# Sigma-1 receptor agonist fluvoxamine for delirium in intensive care units: report of five cases

**DOI:** 10.1186/1744-859X-9-18

**Published:** 2010-04-24

**Authors:** Tsutomu Furuse, Kenji Hashimoto

**Affiliations:** 1 Department of Psychiatry, Asahikawa Red Cross Hospital, Asahikawa, Japan; 2 Division of Clinical Neuroscience, Chiba University Center for Forensic Mental Health, Chiba, Japan

## Abstract

**Background:**

Delirium is a highly prevalent disorder among older patients in intensive care units (ICUs). Although antipsychotic drugs are the medications most frequently used to treat this syndrome, these drugs are associated with a variety of adverse events, including sedation, extrapyramidal side effects, and cardiac arrhythmias. Drug treatment for delirium requires careful consideration of the balance between the effective management of symptoms and potential adverse effects.

**Methods:**

We report on five Japanese men (an 84 year old (acute aortic dissociation: Stanford type A), a 55 year old (traumatic subarachnoid hemorrhage and brain contusion), a 76 year old (sepsis by pyelonephritis), an 85 year old (cerebral infarction), and an 86 year old (pulmonary emphysema and severe pneumonia)) in which the selective serotonin reuptake inhibitor and sigma-1 receptor agonist fluvoxamine was effective in ameliorating the delirium of the patients.

**Results:**

Delirium Rating Scale (DRS) scores in these five patients dramatically decreased after treatment with fluvoxamine.

**Conclusion:**

Doctors should consider fluvoxamine as an alternative approach to treating delirium in ICU patients in order to avoid the risk of side effects and increased mortality from antipsychotic drugs.

## Background

Delirium is a common complication in intensive care units (ICUs) [1-3]. Acute syndrome caused by a disturbance of the cognitive processes in the brain is associated with poor short-term outcomes and may result in adverse sequelae years after ICU discharge [1-3]. Although the pathophysiology of delirium is not fully understood, accumulating evidence suggests that acute oxidative stress responses and inflammation can all contribute to a disruption of neurotransmission (for example, acetylcholine, glutamate, γ -aminobutyric acid, dopamine, serotonin, norepinephrine) and, ultimately, to the development of delirium [1-4]. Antipsychotic drugs are the medications most frequently used to treat this syndrome. However, patients with treated with antipsychotic drugs should be monitored for a variety of adverse events, including hypotension, dystonia, extrapyramidal effects, laryngeal spasm, malignant hyperthermia, glucose and lipid dysregulation, and anticholinergic effects such as dry mouth, constipation, and urinary retention [1-4]. Additionally, there is an association between antipsychotic use (typical or atypical) and increased mortality in older patients [5,6], suggesting that the widespread use of typical and atypical antipsychotic drugs in older adults should be re-evaluated.

The endoplasmic reticulum protein sigma-1 receptors play key roles in Ca^2+ ^signaling and cell survival, and have been shown to regulate a number of neurotransmitter systems in the brain [7-11]. The selective serotonin reuptake inhibitor (SSRI) fluvoxamine is a very potent agonist at sigma-1 receptors, which are also implicated in cognition and the pathophysiology of neuropsychiatric diseases [10,11]. A study using the selective sigma-1 receptor agonist [^11^C]-SA4503 and positron emission tomography demonstrated that fluvoxamine binds to sigma-1 receptors in the living human brain at therapeutic doses, suggesting that sigma-1 receptors might be involved in the mechanism underlying fluvoxamine's action [12].

Given the important role of sigma-1 receptors in the regulation of neurotransmitter systems, we have a hypothesis that fluvoxamine might be effective in the treatment of delirium. Very recently, we reported two cases showing that fluvoxamine was effective in ameliorating the delirium of patients with Alzheimer's disease [13]. Here, we report five cases in which fluvoxamine was also effective in the treatment of delirium in ICU patients.

## Case reports

Table 1 shows the characteristics of five ICU patients with delirium.

**Table 1:** Demographic, clinical, and symptom characteristics of patients with delirium who responded to fluvoxamine

Case	Gender (F/M)	Age (years)	Diagnosis	Dose of fluvoxamine	DRS before treatment	DRS after treatment
1	M	84	Acute aortic association	50 mg	16/32	6/32 (1 day)

2	M	55	Traumatic subarachnoid hemorrhage, brain contusion	50 mg	20/32	10/32 (1 day)

3	M	76	Sepsis by pyelonephritis	50-150 mg	21/32	10/32 (3 days)

4	M	85	Cerebral infarction	50 mg	19/32	10/32 (1 day)

5	M	86	Pulmonary emphysema, severe pneumonia	50-100 mg	18/32	6/32 (2 days)

DRS = Delirium Rating Scale.

### Case 1

An 84-year-old Japanese man was admitted to a hospital's emergency medical center with a complaint of stomach ache. The patient was diagnosed with acute aortic dissociation (Stanford type A) and treated in the ICU. An analgesic effect of pentazocine was observed. However, he had sleep disturbance in the night, and the patient's topic of conversation was inappropriate. Therefore, he was referred to the hospital's department of psychiatry. There, he was disoriented and agitated. To treat his delirium, he was administered fluvoxamine (50 mg, twice a day) and flunitrazepam (1 mg, at night). At 1 day after treatment, his sleep disturbance improved, and his Delirium Rating Scale (DRS) [14] score decreased dramatically from 16/32 to 6/32. After ICU discharge, his condition was good. His Mini-Mental State Examination (MMSE) [15] score was 25/30.

### Case 2

A 55-year-old Japanese man fell from a ladder while working, and a resulting bruise on his head impaired his consciousness. He was admitted to the hospital's emergency medical center. The patient was diagnosed with traumatic subarachnoid hemorrhage and brain contusion by brain computed tomography (CT) and magnetic resonance imaging (MRI). His level of consciousness on the Glasgow Coma Scale [16] in the emergency room was 13/15. His grasp of consciousness was shaky, and he shouted suddenly, whereupon he was referred to the department of psychiatry. He was disoriented, and uttered inappropriate comments. To treat his delirium, fluvoxamine (50 mg, twice a day) and flunitrazepam (1 mg, at night) were added. At 1 day after treatment, his sleep disturbance improved, and his DRS score dramatically decreased from 20/32 to 10/32. After rehabilitation, he recovered gradually. His MMSE score just before discharge was 25/30.

### Case 3

A 76-year-old Japanese man was admitted to a hospital's emergency medical center with a fever of 40°C and in a twilight state. His body CT showed a right kidney stone, and he was diagnosed with sepsis from pyelonephritis. At night time, he became excited and removed his intravenous infusion line, whereupon he was referred to the department of psychiatry. He was disoriented and could not remember why he was in the hospital. A brain CT showed brain atrophy and ventricular enlargement. He had delirium associated with cognitive impairment. To treat the delirium, fluvoxamine (50 mg, twice a day) and flunitrazepam (1 mg, at night) were added. His sleep disturbance improved, but he became agitated. His fluvoxamine dosage was therefore increased to 100 mg and then, the next day, to 150 mg. At 3 days after the start of treatment, his DRS score decreased dramatically from 21/32 to 10/32. His MMSE score just before discharge was 22/30.

### Case 4

An 85-year-old Japanese man was admitted to the emergency medical center with a complaint of languidness on the left side of his body after he fell in the bathroom at his home. The patient was diagnosed with cerebral infarction by brain CT and MRI. At 2 days after hospitalization, he had sleep disturbance and excitation. He was therefore referred to the department of psychiatry. He was also disoriented and agitated. To treat his delirium, fluvoxamine (50 mg, twice a day) and flunitrazepam (1 mg, night) were added. His sleep disturbance improved. At 1 day after this treatment, his DRS score decreased dramatically from 19/32 to 10/32. His MMSE score just before discharge was 22/30.

### Case 5

An 86-year-old Japanese man was admitted to our emergency medical center because he had entered a twilight state after falling in the bathroom at his home. His percutaneous arterial oxygen saturation (SpO_2_) level was 80%, though oxygen was administered with a mask. The patient was diagnosed with pulmonary emphysema and severe pneumonia by chest x-ray and chest CT. To treat the patient's severe pneumonia, antibiotics and steroids were administered. On the night of his fourth day of hospitalization, he became excited suddenly and said that he wanted to go home if other people had contempt for him. He became more excited and booed at a nurse. The next day, he was referred to the department of psychiatry. Although he was not disoriented, he had some delusions; he claimed that a nurse was poisoning his intravenous drip and that a stranger was spying on him. To treat his delirium, fluvoxamine (50 mg, twice a day) and flunitrazepam (1 mg, at night) were added. The next day, his fluvoxamine dosage was increased to 100 mg. At 2 days after the first administration, his DRS score decreased rapidly, from 18/32 to 6/32. At 1 week after the initiation of treatment, his fluvoxamine dosage was reduced to 50 mg. His MMSE score just before discharge (after 4 weeks of hospitalization) was 25/30.

## Discussion

To our knowledge, this report is the first to demonstrate that fluvoxamine is rapidly effective for treating delirium in ICU patients. Nonetheless, a randomized double-blind, placebo-controlled study of fluvoxamine will be needed to confirm its efficacy for the treatment of delirium in ICU patients. Recent findings suggest that sigma-1 receptors might be involved in the different mechanisms of some SSRIs, and that fluvoxamine is a potent sigma-1 receptor agonist [9-11]. Currently, it is unclear whether sigma-1 receptors were involved in the mechanism underlying the beneficial effects of fluvoxamine against the delirium of these ICU patients. Interestingly, donepezil, a potent sigma-1 receptor agonist [17-19], was reported to be effective for treating delirium [20-22], although further studies are necessary. In order to confirm the role of sigma-1 receptors in the treatment of delirium, a randomized double-blind, placebo-controlled study of the selective sigma-1 receptor agonists (for example, cutamesine (SA4503)) in ICU patients with delirium would also be of interest.

Delirium is theorized to be a neurobehavioral manifestation of imbalances in the synthesis, release, and inactivation of a number of neurotransmitters that normally control cognitive function, behavior, and mood [1-4]. At present, it is unclear whether fluvoxamine monotherapy is effective for certain domains of delirium symptoms or for all delirium symptoms equally. Given the role of sigma-1 receptors in the regulation of a number of neurotransmitters, as well as in cognition and mood [7-11], it is likely that sigma-1 receptor agonist may be involved in the fluvoxamine's mechanisms of action, although further study will be necessary.

In this study, in all patients a low dose of flunitrazepam was used for the treatment of insomnia since this drug is considered to be one of the most effective benzodiazepine hypnotics. Therefore, we cannot exclude a possible contribution of flunitrazepam to the efficacy of fluvoxamine for delirium. A further study of fluvoxamine alone will be necessary. In addition, from the present study, we cannot exclude a potential contribution of serotonin transporter inhibition by fluvoxamine to ameliorate delirium in ICU patients. However, it has been reported that the combination of SSRIs with antipsychotic drug(s) and concomitant benztropine might increase the risk of delirium in patients. Byerly *et al*. [23] reported a case showing delirium associated with sertraline, haloperidol and benzotropine. Furthermore, Armstrong *et al*. [24] reported a case of delirium in a patient who was taking benztropine and paroxetine concomitantly. At present, the precise mechanisms underlying the incidence of delirium associated with the combination of sertraline (or paroxetine) and benztropine are currently unclear. Recent findings suggest that sigma-1 receptors might be involved in different mechanisms of some SSRIs [10]. Fluvoxamine is a potent sigma-1 receptor agonist, and sertraline may be a sigma-1 receptor antagonist [10]. Paroxetine has a very low affinity at sigma-1 receptors [10]. Taken together, it is likely that agonism of fluvoxamine at sigma-1 receptors may be involved in the mechanisms of beneficial effects of this SSRI although a further detailed study is necessary.

Drug treatment for delirium requires careful consideration of the balance between the effective management of symptoms and potential adverse effects. As mentioned above, there is an elevated risk of mortality in older patients treated with atypical antipsychotics [5,6], suggesting that the widespread use of atypical antipsychotic drugs in older adults should be re-evaluated. Therefore, the sigma-1 receptor agonist fluvoxamine may serve as an alternative treatment option for older adults with delirium, although further detailed studies on the role of sigma-1 receptors in delirium are necessary.

## Conclusions

These five cases suggest that the sigma-1 receptor agonist fluvoxamine could be an alternative approach to treating delirium in ICU patients because of the risk of extrapyramidal side effects and increased mortality from antipsychotic drugs. More detailed double-blind studies should be performed to clarify the role of sigma-1 receptors in the efficacy of fluvoxamine for delirium in ICU patients.

## Consent

The patients deteriorated mental status made the informed consent procedure reasonably difficult. To this extent consent was obtained from the patient's next-of-kin and effort has been made so that patient identity remains anonymous.

## Competing interests

The authors declare that they have no competing interests.

## Authors' contributions

TF contributed to the clinical and rating evaluations during the follow-up periods. KH conceived of the study and participated in its study and coordination. Both authors read and approved the final manuscript.
